# Mild Concussion, but Not Moderate Traumatic Brain Injury, Is Associated with Long-Term Depression-Like Phenotype in Mice

**DOI:** 10.1371/journal.pone.0146886

**Published:** 2016-01-21

**Authors:** Nikita M. Bajwa, Shina Halavi, Mary Hamer, Bridgette D. Semple, Linda J. Noble-Haeusslein, Mohsen Baghchechi, Alex Hiroto, Richard E. Hartman, André Obenaus

**Affiliations:** 1 Department of Psychology, School of Behavioral Health, Loma Linda University, Loma Linda, California, United States of America; 2 Department of Pediatrics, School of Medicine, Loma Linda University, Loma Linda, California, United States of America; 3 Department of Medicine, Royal Melbourne Hospital, Melbourne Brain Centre, The University of Melbourne, Parkville, Victoria, Australia; 4 Departments of Neurological Surgery and Physical Therapy and Rehabilitation Science, School of Medicine, University of California San Francisco, San Francisco, California, United States of America; 5 Cell, Molecular and Developmental Biology Program, University of California Riverside, Riverside, California, United States of America; 6 Division of Interdisciplinary Studies, School of Behavioral Health, Loma Linda University, Loma Linda, California, United States of America; Uniformed Services University, UNITED STATES

## Abstract

Mild traumatic brain injuries can lead to long-lasting cognitive and motor deficits, increasing the risk of future behavioral, neurological, and affective disorders. Our study focused on long-term behavioral deficits after repeated injury in which mice received either a single mild CHI (mCHI), a repeated mild CHI (rmCHI) consisting of one impact to each hemisphere separated by 3 days, or a moderate controlled cortical impact injury (CCI). Shams received only anesthesia. Behavioral tests were administered at 1, 3, 5, 7, and 90 days post-injury (dpi). CCI animals showed significant motor and sensory deficits in the early (1–7 dpi) and long-term (90 dpi) stages of testing. Interestingly, sensory and subtle motor deficits in rmCHI animals were found at 90 dpi. Most importantly, depression-like behaviors and social passiveness were observed in rmCHI animals at 90 dpi. These data suggest that mild concussive injuries lead to motor and sensory deficits and affective disorders that are not observed after moderate TBI.

## Introduction

Traumatic brain injury (TBI) is an insult to the brain caused by an external physical force, presents a serious and emerging medical problem and is the leading cause of death and disability both in combat and civilian populations. Falls are the leading cause of TBI in the United States (U.S.) [1], but other causes include motor vehicle accidents, sports injuries [2–4], and violence [5]. An estimated 1.7 million individuals are affected by TBI annually in the U.S., with a substantial number of deaths and permanent disabilities [5,6]. The U.S. military has estimated that 22% of all combat wounds in Iraq and Afghanistan were brain injuries, and TBI is now referred to as the “signature wound” of these recent military conflicts [5,7,8]. This phenomena impacts all ages and socioeconomic classes and can result in acute and/or delayed motor, cognitive[9], emotional deficits [10], and reduced quality of life [11]. Thus, TBI is a major public concern, and other than palliative care, treatment options remain limited [12].

TBI can be classified by severity (e.g., mild, moderate, or severe) based on the length of time of lost consciousness [5] and standard clinical imaging techniques, such as magnetic resonance imaging (MRI) [3] and diffusion tensor imaging [13,14]. Mild TBI (mTBI; concussion) accounts for 75% of all TBIs in the U.S. each year [15]. mTBI can cause cognitive, physical, and affective deficits that lead to social problems, disability and/or unemployment [16–18]. Based on the severity of the brain injury and other factors, such as the injury location, cohort heterogeneity, and time since the injury, a wide variety of deficits may occur following TBI. In one study, 38% of TBI patients developed a clinically significant mood disorder within six months of injury [19]. A more recent study reported depression as the most common neuropsychiatric consequence of TBI, with a frequency of 25–50% and a continued increased risk of developing depression even decades following the TBI [20]. They also reported that depressive disorders following TBI are associated with high levels of anxiety and apathy, leading to diminished goal-directed behaviors such as effort, initiative, and productivity. Reports also show aggressive behavior in TBI patients significantly correlates with major depression and poor social functioning [21].

The risk of experiencing more severe brain injuries and the resulting behavioral deficits increases with each repeated mTBI (rmTBI) [22]. Individuals who have experienced one brain injury are three times as more likely to experience a second brain injury [23]. Repeated brain injuries have been found to worsen tissue integrity [13,14], increase physiological changes [3], and increase behavioral deficits significantly more than a single impact [22,24]. An understanding of rmTBI’s pathophysiology and mechanisms is necessary to prevent and treat such outcomes in those at high risk for rmTBI, such as athletes [2,14,22].

Experimental models of TBI are categorized as open- or closed-head, depending on whether the skull is penetrated [25]. These models have been developed and well characterized to examine pathologies ranging from mild to severe [26–29], with symptoms including, but not limited to significant motor, behavioral [26,30–33], affective [24,34,35], and social disturbances [36]. One of these models, controlled cortical impact (CCI), is a commonly used rodent model of TBI that approximates clinical brain injury in which a direct piston impact to the cortex induces a number of acute and long-term behavioral impairments. The degree of injury can be manipulated by varying the piston’s speed and/or depth, where more severe injuries generally induce larger locomotor and cognitive deficits [25,37].

We recently used CCI to investigate the temporal development of neuropathology using MRI to assess the effects of rmTBI by administering 1 impact to each cortical hemisphere [38]. We found that tissue damage was exacerbated following the second mTBI, especially when the injuries were seven days apart. Our studies suggest that the brain remains vulnerable to a subsequent injury for a period of time, increasing the probability of behavioral deficits. We extended these studies to investigate white matter and found abnormalities as late as 60 days post injury (dpi) [39]. Similarly, we showed that brain injury early in life induced long-term white matter abnormalities, delayed development, and persistent behavioral deficits [40].

Rodent models of closed-head injury (CHI) have been developed to mimic mTBI/concussive conditions observed clinically, with the aim to elicit a global brain injury through rotational stress caused by head movement [22,27]. CHI models vary in methodology and severity, with pathologies ranging from mild to severe [24,27,28,41,42] and current attention has been drawn to the consequences of repetitive brain injury. Mice exposed to repeated mild CHI (rmCHI) with three weight-drop sessions 24 hours apart experienced prolonged loss of consciousness and impaired water maze spatial learning performance [43]. Another rmTBI model, in which repeated impact to the cranium was delivered to an unrestrained mouse, induced hyperactivity and motor coordination deficits [44]. An rmTBI model in which rats were given 1, 3, or 5 mild lateral fluid percussion injuries spaced five days apart induced short (24 hr) and long-term (8 weeks) cognitive impairments [45]. Specifically, five mild repeated concussive injuries elicited increased anxiety and depression-like symptoms. In another rmTBI model, mice received six impacts per day for seven consecutive days presented with depression-like symptoms at 30 dpi [24]. Mice exposed to a single mTBI weight drop session to the temporal region of the brain experienced impaired learning and memory, as well as depression-like symptoms up to 90 dpi [46,47].

Long-term affective deficits have not been investigated following repeated mild concussions in mice to model similar deficits that are often observed in humans with repeated concussions [11,18,20,22]. In this study, we assessed affective, social, learning, and motor behaviors in mice following mild closed head injury (mCHI) or repeated mCHI (rmCHI) at acute (1, 3, 5, and 7 dpi) and long-term (90 dpi) time points. A moderate single CCI cohort was used as a positive control to confirm deficits that result from a moderate TBI.

## Material and Methods

### Animals

**Fig 1:**
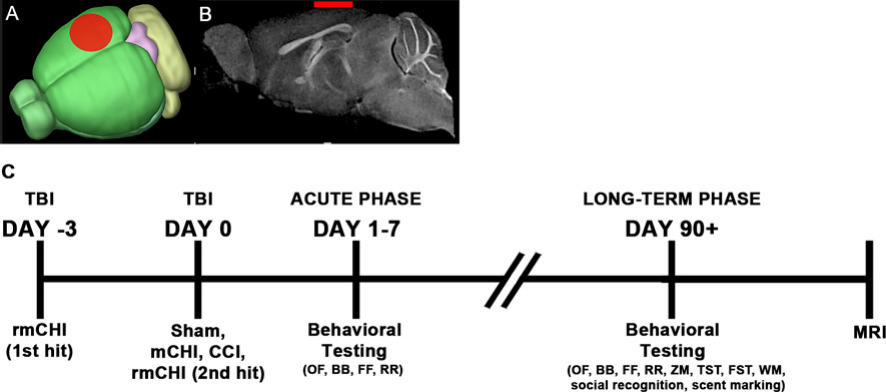
Experimental design of the study. **(A)** CHI mice were impacted in the right hemisphere (red), followed by an identical impact in the left hemisphere in rmCHI mice. **(B)** A representative image of the brain and injury site (red bar) after impact in our CHI model. No overt tissue damage is observed after injury. **(C)** Mice underwent neurological and behavioral testing in the acute phase (1–7 dpi) and also at a chronic phase (90 dpi). Testing in the acute phase consisted of the open field (OF), balance beam (BB), foot fault (FF), and rotarod (RR). Testing at 90 dpi consisted of the same acute tasks above, as well as the elevated zero maze (ZM), water maze (WM), tail suspension test (TST), forced swim test (FST), social recognition test, and scent-marking test. At the end of behavioral testing, animals underwent perfusion fixative for high resolution MRI.

All protocols and procedures were approved by the Institutional Animal Care and Use Committee of Loma Linda University and comply with the principles and procedures of the Guidelines for the Care and Use of Experimental Animals. Adult 3-month-old male C57BL/6J mice (Jackson Laboratory, Bar Harbor, ME) were housed individually in cages on a 12-hr light-dark cycle at constant temperature and humidity, and fed *ad libitum*. All experimental animals were randomly assigned to groups that included sham (anesthesia only; n = 10), moderate CCI (n = 10), mCHI (n = 10) or rmCHI (1 to each hemisphere spaced 3 days apart; n = 10). One rmCHI mouse died during anesthetic recovery, and all other mice survived through 90 days post injury. Details of the experimental design are described in text (Fig 1).

### Controlled cortical impact (CCI) injury

We used a moderate CCI with a 1.0 mm depth modeled after Yu et al. [48]. In their study, cortical tissue loss was accompanied by significant motor, learning and memory deficits [48]. CCI was induced in adult mice similar to that previously described in rats [38]. Briefly, mice were anesthetized (isoflurane: 3% induction, 1–2% maintenance; Webster Veterinary Supply, Inc., Sterling, MA) and secured in a mouse stereotaxic frame (David Kopf Instruments, Tujunga, CA) with a heating pad that maintained body temperature at 37°C. Anesthesia was monitored throughout the surgery. Lidocaine (0.01 mg/kg; dilution: 0.01 mg/mL) was injected subcutaneously at the scalp incision for pain relief from the surgical procedure. Following a midline incision over the skull, a 5 mm craniotomy was performed over the right temporal-parietal cortex. The center of the injury was located 2.5 mm below Bregma and 2.5 mm to the right of the Sagittal midline. CCI was induced using an electromechanical impactor (3-mm diameter metal tip) and centered over the exposed dura at an 18° angle, perpendicular to the cortical surface (Leica Microsystems Company, Richmond, IL). The CCI was delivered to a 1.0 mm depth directly into the cortical surface with impact duration of 200 milliseconds at a velocity of 5 m/sec, resulting in moderate cortical injury. The surgical site was sutured after recording any bleeding or herniation of cortical tissues. Additional buprenorphine was applied along the sutures for post-surgery pain relief before the animals were returned to their home cages.

### Mild closed head injury (mCHI)

Mice were anesthetized with isoflurane (isoflurane: 3% induction, 1–2% maintenance) and placed on a cushioned foam base (Foam to Size Inc., Ashland, VA). Lidocaine (0.01 mg/kg; dilution: 0.01 mg/mL) was injected subcutaneously for post-surgery pain relief from the surgical procedure. Following a midline incision and retraction of the skin overlying the skull, injury was induced to the right temporal-parietal region of the skull using a 4-mm rounded plastic tip (Delrin) affixed to a nitrogen driven pneumatic impactor. The center of the injury was located 2.5 mm below Bregma and 2.5 mm to the right of the Sagittal midline. The injury was delivered using impact duration of 250 milliseconds at 40 psi and resulted in a rotational head displacement of 1 mm. This impact to the skull resulted in a mild rotational injury, compared to the invasive nature of CCI that impacted cortical tissue directly and caused more damage. The surgical site was sutured after impact and no bleeding along skull sutures or fractures were observed in the mice. During model development high-speed video confirmed the rotational nature of the impact and that parameters used in this study did not result in any skull fractures or bleeding of the sutures. Additional buprenorphine was applied along the sutures for post-surgery pain relief before the animals were returned to their home cages. Repeated mCHI (rmCHI) mice underwent similar surgical procedures with an additional injury over the left temporal-parietal cortex 3 days after the initial impact. The center of the injury was located 2.5 mm below Bregma and 2.5 mm to the right of the Sagittal midline.

### Behavioral testing

Behavior was tested acutely at 1, 3, 5, 7 dpi and again at 90 dpi. All behavioral tests at each time point were carried out within the 12-hr-light cycle. Groups were interleaved in the testing sequence and experimenters were blinded to the treatment groups. Acute tests administered on 1, 3, 5, and 7 dpi were completed in the following order: open field, balance beam, foot fault, and rotarod. Behavioral tests starting at 90 dpi included those given in the acute phase, followed by the elevated zero maze, tail suspension, forced swim, water maze, social recognition test, and the scent marking test. Testing began at 90 dpi and continued consecutively for 10 days (90+).

#### Open field activity

Open-field testing assessed general exploratory behavior and activity levels [49]. Mice were placed in a 49 cm x 36 cm opaque open-topped plastic bins and allowed to explore, unrestricted, for the duration of 30 minutes. Movements of each animal were recorded by an overhead camera and analyzed by a computerized tracking system (Noldus Ethovision; Information Technology, Inc., Leesburg, VA). Total distance traveled was assessed as a measure of overall activity level.

#### Balance beam

Fine motor coordination and balance was assessed using the balance beam test [50]. A square acrylic glass balance beam (61 cm long, 0.65 cm wide) labeled in 2.5-cm increments was used. Mice were placed at the midpoint of the beam, perpendicular to the longitudinal axis. They were allowed to walk unrestricted in either direction for 60 seconds, with two trials 30 minutes apart. The number of falls, total time spent on the beam, distance traveled, left and right turns, and the numbers of left or right paw slips were recorded.

#### Foot fault

Foot fault testing was carried out on an elevated wire mesh (2.5 x 30 cm rectangular holes / grid spacing) raised 76 cm above the floor. Mice were placed in the middle of the wire mesh and their movements were both manually and video-recorded for a period of 60 seconds in two separate trials 30 minutes apart [51]. When a mouse paw slipped completely through the wire mesh, it was considered an individual foot fault. The average foot fault score was calculated from the total number of faults from the two separate trials.

#### Rotarod

The accelerating rotarod is a test of sensorimotor coordination and balance [52] that consists of a 3-cm diameter rotating horizontal cylinder (Rotamex-5; Columbus Instruments, Columbus, OH). Mice were placed on the cylinder and had to continuously walk forward to avoid falling. Latency to fall was recorded [52,53]. Mice were tested with three blocks of two consecutive trials per day. Blocks consisted of two stationary trials (at 5 RPM steady), two trials that started at 5 RPM and accelerated by 3 RPM every 5 seconds, and two trials that started at 5 RPM and accelerated by 3 RPM every 3 seconds. Each trial lasted up to 60 seconds with approximately 45–60 minutes between each block. Performance over days of testing is a measure of motor learning.

#### Elevated zero maze

The elevated zero maze was used to assess exploratory behaviors in an anxiety-provoking environment [54]. The maze consisted of a plastic 100 cm outer diameter ring, with a 10 cm wide channel and the two opposing quadrants were enclosed with 35 cm walls. The room was dimmed, and halogen lights directly illuminated the open spaces of the maze. Animals were placed in the center of one of the open spaces and allowed to freely explore the zero maze for 5 minutes. The percentage of time spent in the enclosed quadrants was calculated. Spending more time in the enclosed spaces is generally associated with anxiety-like behavior, whereas increased time in the open quadrants may be associated with increased risk-taking behaviors.

#### Tail suspension test

The tail suspension test was administered to assess depression-like behaviors [55]. Mice were suspended by the tail with adhesive tape that was attached approximately 1 cm from the tip of the tail. The other end of the tape was wrapped around a hook that was embedded in the center of the ceiling of a wooden box (19 cm x 21 cm x 40 cm). Once suspended, the animal’s head end was approximately 20 cm from the floor of the box. The box was enclosed on all sides except for the viewing side, and lighting and sound in the room were kept at a minimum. Each animal received one 6-min trial and in which mobility and agitation (struggling) were recorded for the duration of 6 minutes. The amount of time that the animal remained immobile during the final 4 minutes of the trial was reported. Immobility was defined as the complete lack of movement by the mouse, even if it was still swinging back and forth from a previous struggle or if it was curled up while holding its paws (as long as it was not struggling or moving otherwise).

#### Forced swim test

The forced swim test was also administered to assess depression-like behaviors [56]. Mice were placed in glass cylinders (21 x 12 cm) containing 12 cm of water (22–25°C) for 6 minutes. White cardboard enclosed the cylinders on all sides except for the viewing side, and the sound in the room was kept at a minimum. Each animal received one 6-min trial. Assistants, blinded to the treatment groups, individually rated the mouse on mobility and escape behavior for the trial duration. The time that the animal remained immobile during the final 4 minutes of the trial was recorded. Immobility was defined as either a complete lack of movement or gradually pedaling with hind legs to remain afloat.

#### Water maze

Learning and memory were assessed water maze spatial navigation [57,58], which requires an animal to learn the location of a hidden platform in a pool of water using the visual cues from around the room. The water maze consisted of a metal pool (110 cm diameter) filled with water that is colored opaque with white tempura paint. The pool contains a moveable platform (11 cm diameter) that the animal can step onto to escape the water. Animals were given a total of 10 trials per day for 5 consecutive days. For each trial, an animal was released into the pool, with its nose against the wall at one of the four release points and allowed to swim to the platform. The trials lasted a maximum of 60 seconds. If the mouse did not find the platform in the allotted time, it was manually guided to the platform. An overhead camera recorded the swim paths, which gathered data for the quantification of distance, latency, proximity to target, and swim speed by a computerized tracking system (Noldus Ethovision). Cued learning, which is a control task for assessing sensorimotor and/or motivational deficits that may affect performance during the spatial phase, was assessed on day 1 of the water maze protocol. The surface of the escape platform was visible (5 mm above the surface of the water) and a pole was placed on top of the platform to make its location more obvious. The location of the platform varied from trial to trial. Animals were released into the pool opposite the location of the platform and were allowed to remain on the platform for 5 seconds after finding it. As performance improves, escape latency and swim path length generally decrease.

Spatial learning was assessed on days 2 and 3 of the water maze protocol. In this phase of testing, the mice had to find the platform based on its relationship to the spatial cues around the room, rather than direct visualization. The escape platform was submerged 1 cm below the surface of the opaque water, and the location of the platform changed each day. After finding the platform, animals were allowed to remain there for an additional 5 seconds. A probe trial was administered on day 3. In the probe trial, the platform was removed from the pool, allowing the animal to search for the platform for 60 seconds. The amount of time the animal spent in the probe quadrant was measured as well as the total number of times the animal crossed over the former location of the platform. An hour later, the platform was placed back into the pool at a new location, and the next sets of 10 trials were administered.

Spatial working memory was assessed on days 4 and 5 of the water maze protocol. In this phase of testing, the escape platform was submerged 1 cm below the surface of the opaque water, and the location of the platform changed after each block. After finding the platform, animals were allowed to remain on there for an additional 5 seconds. Since the platform’s location was unknown to the mouse on the first trial of each block, improved performance on the second trial of each block reflected better spatial working memory. Probe trials were also administered on days 4 and 5, before each working memory paradigm.

#### Social recognition test

The social recognition test was administered to assess social interaction and memory [36]. Mice were removed from their home cages and placed in a novel cage, with bedding and a filter cage top, without a wire food hopper. During testing, an unfamiliar male mouse was used as a stimulus object, and was placed into the cage with the test mouse for 1-minute interaction/habituation sessions. Both the test and stimulus mice were allowed to move around the cage freely during testing. The same stimulus mouse was placed in the cage of the test mouse repeatedly for 3 trials. In the 4^th^ trial, a novel stimulus mouse was introduced into the cage of the test mouse for 1 minute. Mice were tested in groups of five, so that each 1-minute interaction was separated by approximately 5 minutes of rest.

Experimenters recorded time spent in active social contact performed by the test mouse. Social contact initiated by the stimulus mouse was not included in the time measures. Behaviors that were scored as interaction were sniffing with the nose within 1 cm of the stimulus mouse (including nose, body, and anogenital area), pawing and climbing on the stimulus mouse, time mice spent side by side and any aggressive behavior defined as biting, clawing, and fighting (i.e. using forelimbs to hit) were recorded [36].

#### Scent marking test

Scent marking behaviors in mice are generally used in a context-dependent manner, to distinguish territories, recognize individuals, communicate behavior and attract mates [36,59]. In this test, we recorded urinary pheromone traces and fecal boli in response to the presence of a novel female mouse. The open field area (30.5 cm x 30.5 cm; custom made) was lined with paper at the base, with an inverted porous plexiglass cylinder (6.3 cm x 10.3 cm; custom made) placed in the middle. Baseline recordings were acquired followed by testing with a stimulus female mouse the next day. Female mice were placed inside an inverted cylinder and males were introduced to the arena. In both testing conditions, male mice were allowed to explore, unrestricted, for 20 minutes. Movements were recorded by an overhead camera and analyzed by a computerized tracking system (Noldus Ethovision). Time and distance in the center and periphery of the open field area were analyzed. The center boundary was drawn as a circle around the cylinder at a width of ~8 cm (mouse body length) and kept consistent across animals. Paper at the base of the testing area was removed and set to dry completely before spraying with ninhydrin solution (Sigma-Adlrich, St. Louis, MO) to detect scent markings. After each task, the number of fecal boli was also recorded. Each paper was digitized and analyzed using Image Pro. Scent markings positively detected with the ninhydrin solution were subtracted from the background and scored. Increased scent marking in the presence of the stimulus female indicates more dominant and social behavior by male mice [36,59].

### Ex vivo magnetic resonance imaging

**Fig 2:**
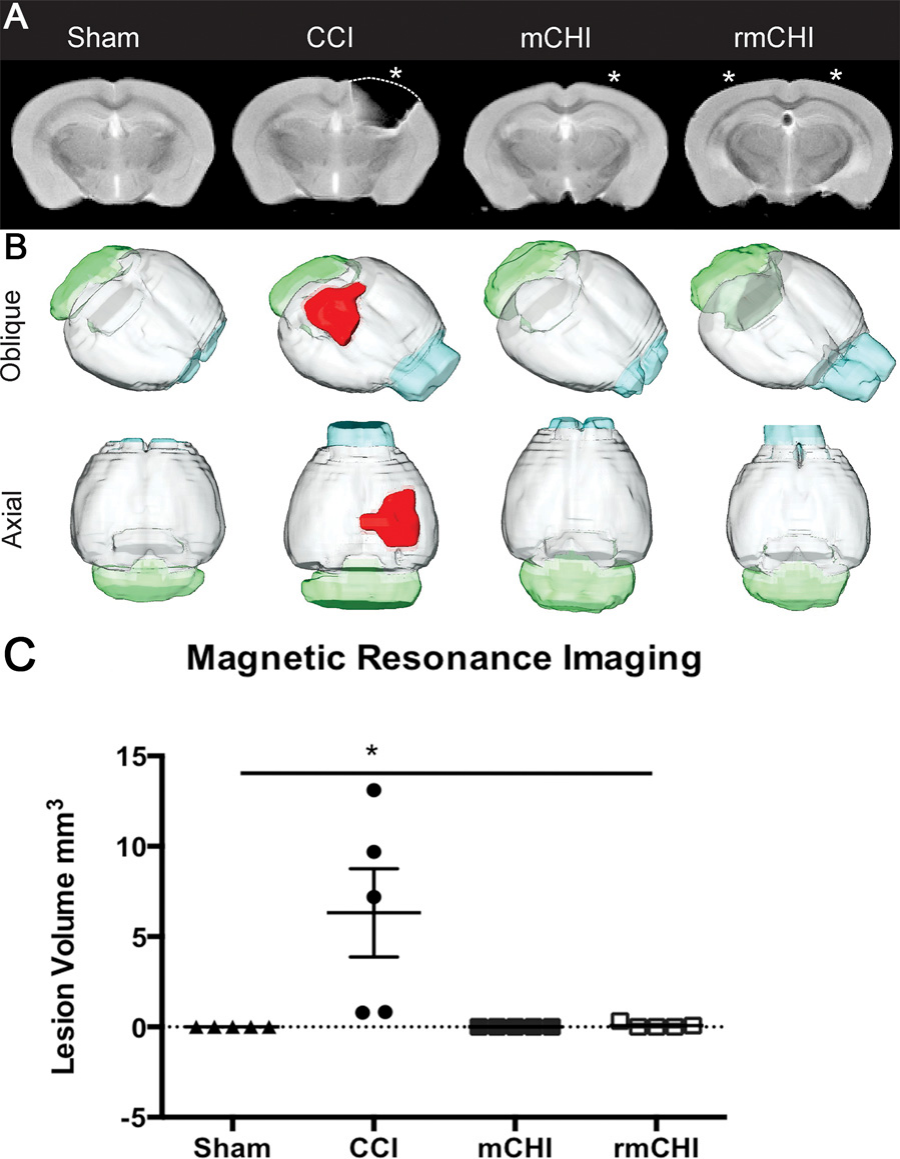
MRI assessment of severity and volumetric analysis (A) T2-weighted MRI at the level of the injury site (*) revealed no overt brain damage in sham, mCHI or rmCHI mice. In contrast, mice subjected to a moderate CCI exhibited a visible lesion within the cortex. (B) 3-D reconstruction of the T2-weighted MRI of the brain revealed no overt cortical damage in sham, mCHI or rmCHI mice. Mice subjected to a moderate CCI revealed a visible lesion (red) within the cortex. (C) MRI analysis revealed significant tissue loss in CCI mice compared to sham, mCHI and rmCHI at 90 dpi (*p* < .01).

High resolution MRI was performed *ex vivo* at 90 dpi as previously described [60]. Animals were sacrificed via transcardial perfusion with 10% formalin prepared in phosphate-buffered saline (PBS). Extracted brains were immersed in the same solution overnight, and then transferred to PBS before *ex vivo* scanning. Five representative animals per group were selected to undergo structural T2 weighted MRI to determine lesion volumes visible following CCI and CHI (Fig 2). T2 weighted images (T2WI; TR/TE = 3500 ms/10 ms/10 echos, 50x0.5 mm slices) were collected using a 256x256 matrix on a 9.4T Bruker Avance instrument (Bruker Biospin, Billerica, MA). Quantitative T2 maps were computed from T2WIs using in-house software written in Matlab (Mathworks, Natick, MA) as we have previously described [60].

The TBI lesion was manually drawn on each T2WI slice based on hyper- or hypo-intense regions relative to normative brain tissues using Cheshire image processing software (Hayden Image/Processing Group, Waltham, MA). Whole brain analysis spanned the entire cerebrum starting where the olfactory bulbs merged into the cortex to the last MRI slice that contained cortical tissues. The brainstem was not included in the whole brain volume calculations. Lesion and whole brain areas for each slice were brought into Excel for final volume calculations. Finally, 3-D volumetric renderings for representative mice were produced using Amira (FEI, Hillsboro, OR).

### Statistical analysis

Statistical analyses used an α-level of 0.05 for tests of significance (IBM SPSS Statistics 21.0). Correlations between behavioral variables were determined using the Pearson product-moment coefficient. Data from all behavioral testing were analyzed with repeated measures and one-way ANOVAs. Interactions between factors are reported if statistically significant. Tukey’s HSD tests were used to post-hoc comparisons. Swim data from the water maze were analyzed by averaging trials into 5 blocks of 2 trials each. These blocks were analyzed with repeated measures ANOVAs that included one within-subjects variable (test day). If any assumptions of compound symmetry and sphericity were violated, the reported *p*-values for each repeated-measures analysis reflected the Huynh-Feldt adjustment to the degrees of freedom.

## Results

There were no significant weight differences between the injury groups across time in our study, with an average final weight at 90 dpi of 34.93 g, SD = +/-2.96 (sham = 35.26 g, SD = +/-3.25; CCI = 34.33 g, SD = +/-2.37; CHI = 34.56 g, SD = +/-3.89; rmCHI = 35.65 g, SD = +/-2.26).

### Lesion Analysis from MRI

Five representative animals per group underwent structural MRI at 90 dpi. mCHI and rmCHI mice had no overt lesions, in contrast to the significant moderate brain lesion observed in CCI mice (Fig 2A; compare sham to mCHI or rmCHI), confirming the mild concussive nature of the CHI model. The 3-D reconstruction of the T2-weighted images further illustrates the lack of cortical damage in the mCHI and rmCHI group compared to that found in CCI mice (Fig 2B). The cortical tissue loss caused by CCI corresponds to the changes in behavior, particularly motor effects. CCI mice had significant tissue loss at the site of impact in CCI mice (F_3, 16_ = 6.65, < .01; Fig 2C). With our mild concussion model, the behavioral effects observed are not influenced by brain volume changes or lesion size in CHI or rmCHI animals.

### Acute and Long-term Motor Effects

**Fig 3:**
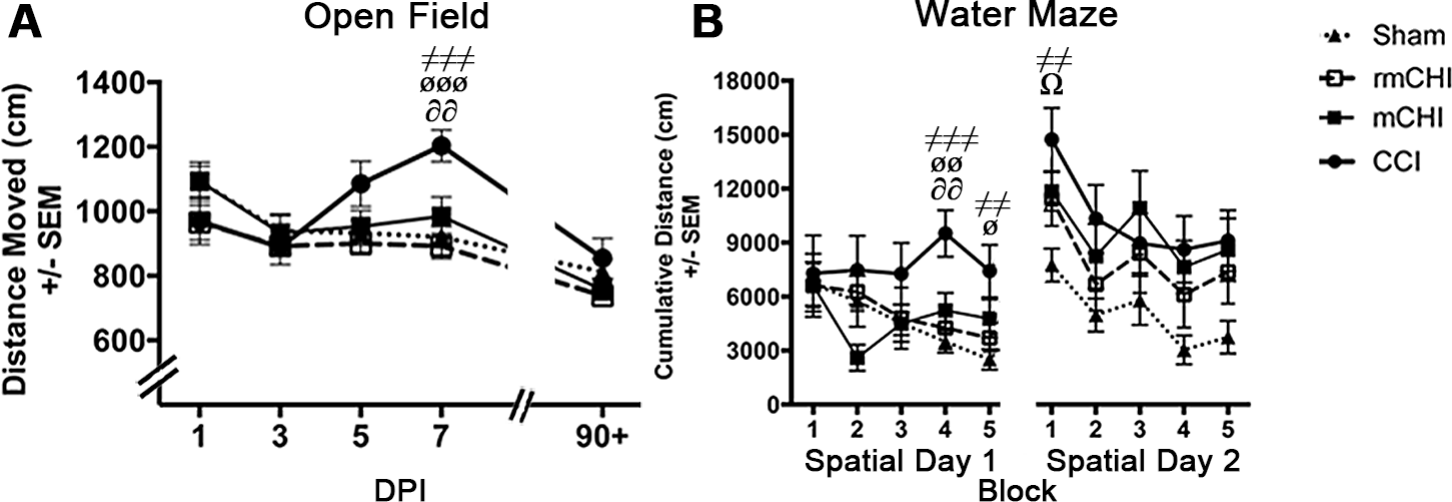
Moderate CCI induced hyperactivity and spatial learning deficits. **(A)** Moderate CCI mice exhibited hyperactivity in the open field, traveling a significantly greater distance at 7 dpi compared to sham (^≠≠≠^*p*<0.001), rmCHI (^øøø^*p*<0.001), and mCHI (^∂∂^*p*<0.01). CCI-induced hyperactivity was not observed at 90+ dpi. **(B)** At 90+ dpi, CCI mice exhibited severe deficits in the spatial water maze task, with no evidence of learning the escape platform’s location on day 1. These learning deficits persisted when the platform’s location was changed on day 2. (^≠≠^*p*<0.01 for CCI compared to sham, ^≠≠≠^*p*<0.001 for CCI compared to sham, ^ø^*p*<0.05 for CCI compared to rmCHI, ^øø^*p*<0.01 for CCI compared to rmCHI, ^∂∂^*p*<0.01 for CCI compared to mCHI, ^Ω^*p* < .05 for mCHI compared to sham).

**Fig 4:**
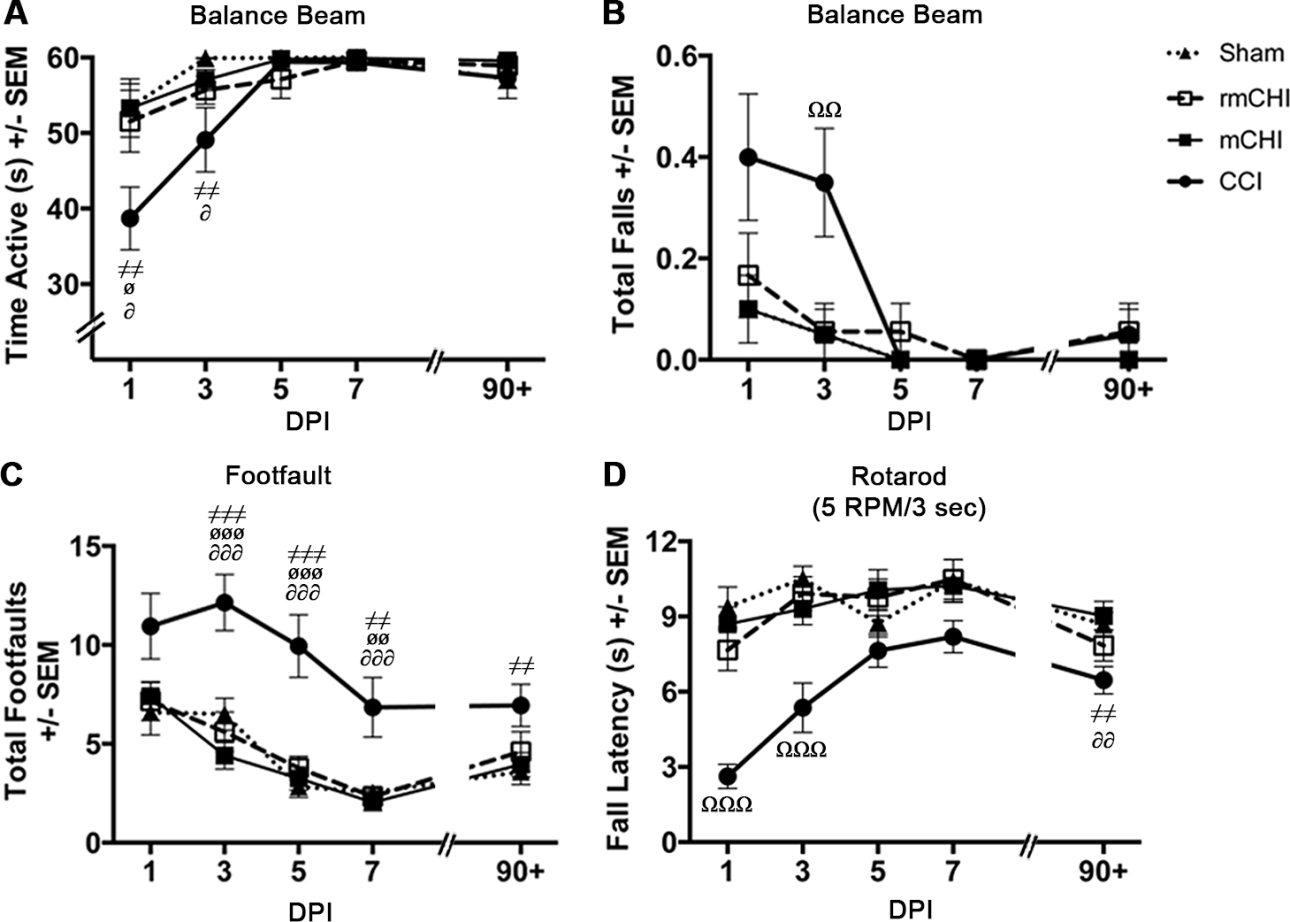
Motor deficits are evident in the CCI, but not mCHI or rmCHI mice. **(A)** At 1 dpi, CCI mice spent significantly less active time on the beam than sham (^≠≠^*p*<0.01), rmCHI (^ø^*p*<0.05), and mCHI (^∂^*p*<0.05) mice. They also spent significantly less time being active on the beam at 3 dpi than sham (^≠≠^*p*<0.01) and mCHI (^∂^*p*<0.05). **(B)** At 3 dpi, the CCI mice fell off the balance beam significantly more often than the other injury groups (^ΩΩ^*p*<0.01). **(C)** rmCHI and mCHI mice exhibited no deficits on the foot fault task, but CCI mice had more foot faults than shams, rmCHI, and mCHI mice at 3 dpi (^≠≠≠^*p*<0.001, ^øøø^*p*<0.001, and ^∂∂∂^*p*<0.001, respectively), 5 dpi (^≠≠≠^*p*<0.001, ^øøø^*p*<0.001, and ^∂∂∂^*p*<0.001), and 7 dpi (^≠≠^*p*<0.01, ^øø^*p*<0.01, and ^∂∂∂^*p*<0.001). CCI mice still had significant deficits at 90+ dpi versus shams (^≠≠^*p*<0.01) and mCHI (^∂^*p*<0.05) mice. **(D)** CCI, but not mCHI or rmCHI, mice performed significantly worse on the accelerating rotarod test (5 RPM every 3 sec) at 1 and 3 dpi (^ΩΩΩ^*p*<0.001). These deficits were still present at 90+ dpi compared to shams (^≠≠^*p*<0.01) and mCHI mice (^∂∂^*p*<0.01).

**Fig 5:**
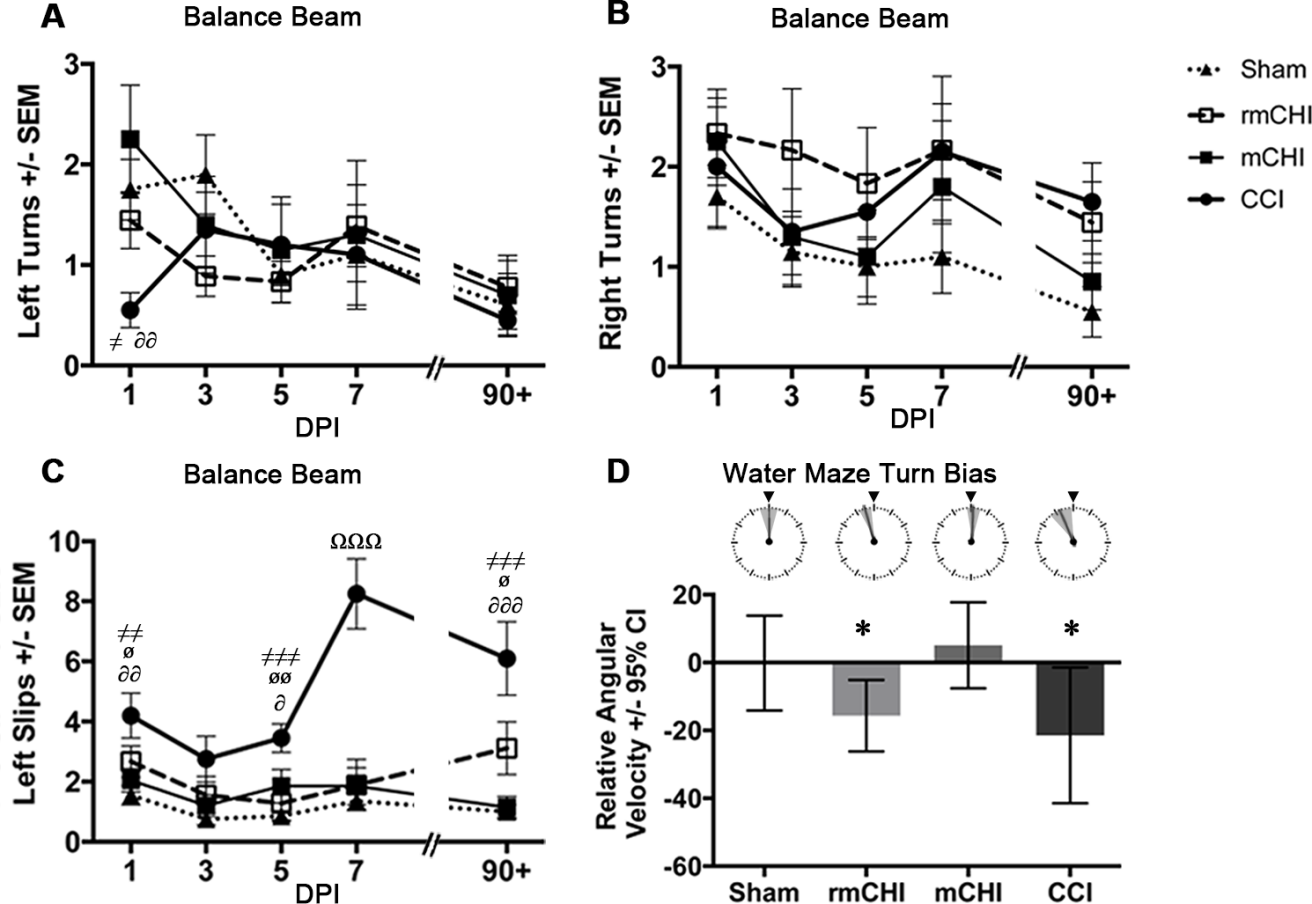
Turn bias was most evident in the mice with CCI. **(A)** At 1 dpi, CCI mice made significantly fewer left turns (contralateral to the injury) on the balance beam than sham (^≠^*p*<0.05) and mCHI (^∂∂^*p* < .01) mice. **(B)** Both rmCHI and CCI mice exhibited more turns to the right throughout balance beam testing than shams, though these results did not reach statistical significance. **(C)** CCI mice made more left-sided slips on the balance beam than other animals. These deficits were most prominent at 7 dpi compared to shams and other injury groups (^aaa^*p*<0.001) and persisted up to 90+ dpi (^≠≠^*p*<0.01 vs. sham, ^≠≠≠^*p*<0.001 vs. sham, ^ø^*p*<0.05 vs. rmCHI, ^øø^*p*<0.01 vs. rmCHI, ^∂^*p*<0.05 vs. mCHI, ^∂∂^*p*<0.01 vs. mCHI, ^∂∂∂^*p*<0.001 vs. mCHI). **(D)** In the water maze, rmCHI and CCI mice tended to swim to the left (contralateral to the injury) compared to sham and mCHI animals (*p<0.05, **p<0.01; mean +/- 95% CI). 95% confidence intervals show that the left turn biases in rmCHI and CCI mice are statistically significant (*p*<0.05), whereas no significant turn bias is observed in the sham and mCHI mice. (The clock graphic above shows variance markings of the relative angular velocity of each injury group with each mark on the clock representing a separation of 6° in relation to 12 o’clock. Flag symbol marks zero degrees).

CCI mice were hyperactive in the open field at 7 dpi compared to sham (*p* < .001), mCHI (*p* < .01), and rmCHI (*p* < .001) mice, though this effect had dissipated by 90 dpi (F_3, 35_ = 9.04, *p* < .001; Fig 3A). mCHI and rmCHI mice behaved similarly to shams in the open field. The balance beam revealed acute motor coordination and balance deficits in CCI, but not mCHI or rmCHI, mice. CCI mice were the least active on the beam at 1 (F_3, 35_ = 3.52, *p* < .05; Fig 4A) and 3 dpi (F_3, 35_ = 3.57, *p* < .05); Fig 4A) and at 3 dpi, fell from the beam more than sham and the CHI groups (F_3, 35_ = 4.55, *p* < .01; Fig 4B). All injured groups tended to make more right turns throughout balance beam testing, though these results did not reach statistical significance (Fig 5B). In contrast, CCI mice made fewer left turns on the beam at 1 dpi (F_3, 35_ = 3.52, *p* < .05 vs. mCHI; Fig 5A). CCI mice also slipped on the beam’s left side more than other groups. This deficit was most prominent at 7 dpi (F_3, 35_ = 17.46, *p* < .001) and persisted through 90 dpi (F_3, 35_ = 9.60, *p* < .01) compared to sham (*p* < .001), mCHI (*p* < .001) and rmCHI mice (*p* = .05; Fig 5C). A less prominent left-sided slip deficit also emerged in the rmCHI group at 90 dpi (Fig 5C).

mCHI and rmCHI mice exhibited no deficits on the foot fault task (Fig 4C), but CCI mice had an increased number of foot faults compared to shams, mCHI, and rmCHI mice at 3 dpi (~120%; compared to shams; F_3, 35_ = 11.63, *p* < .001), 5 dpi (~205%; F_3, 35_ = 12.68, *p* < .001), and 7 dpi (~195%; F_3, 35_ = 7.72, *p* < .001). CCI mice still had significant deficits at 90 dpi (F_3, 35_ = 3.24, *p* < .05) versus shams (*p* < .01) and mCHI (*p* < .05) mice. Finally, CCI, but not mCHI or rmCHI, mice fell off the accelerating rotarod more quickly at 1 (F_3, 35_ = 19.36, *p* < .01) and 3 dpi (F_3, 35_ = 10.57, *p* < .01; Fig 4D). These deficits were still present at 90 dpi (F_3, 35_ = 4.39, *p* < .05) compared to shams (*p* < .01) and mCHI mice (*p* < .01; Fig 4D).

### Long-term Learning Effects

CCI mice exhibited severe spatial learning deficits in the water maze (Fig 3B) when tested at 90 dpi, showing no evidence of learning the escape platform’s location on day 1 as indicated by block 4 (F_3, 35_ = 6.73, *p* < .01) compared to sham (*p* < .001), mCHI (*p* < .01), and rmCHI (*p* < .01) mice and block 5 (F_3, 35_ = 3.67, *p* < .05) compared to sham (*p* < .01) and rmCHI (*p* < .05). All injury groups performed worse than shams when the platform’s location was changed on day 2, but only the CCI group’s deficit on block 1 attained significance compared to shams (F_3, 35_ = 4.53; *p* < .01; Fig 3B). In probe trials, CCI mice failed to exhibit a bias toward the previous day’s platform quadrant (F_3, 35_ = .78, *p* = .52). Swim analysis (F_3, 35_ = 2.81, *p* = .05) also revealed that CCI mice swam slower compared to sham (*p* < .05; data not shown), and 95% confidence intervals show that both CCI and rmCHI mice had a significant tendency to drift to the left (*p* < .05; Fig 5D).

### Long-term Affective Effects

**Fig 6:**
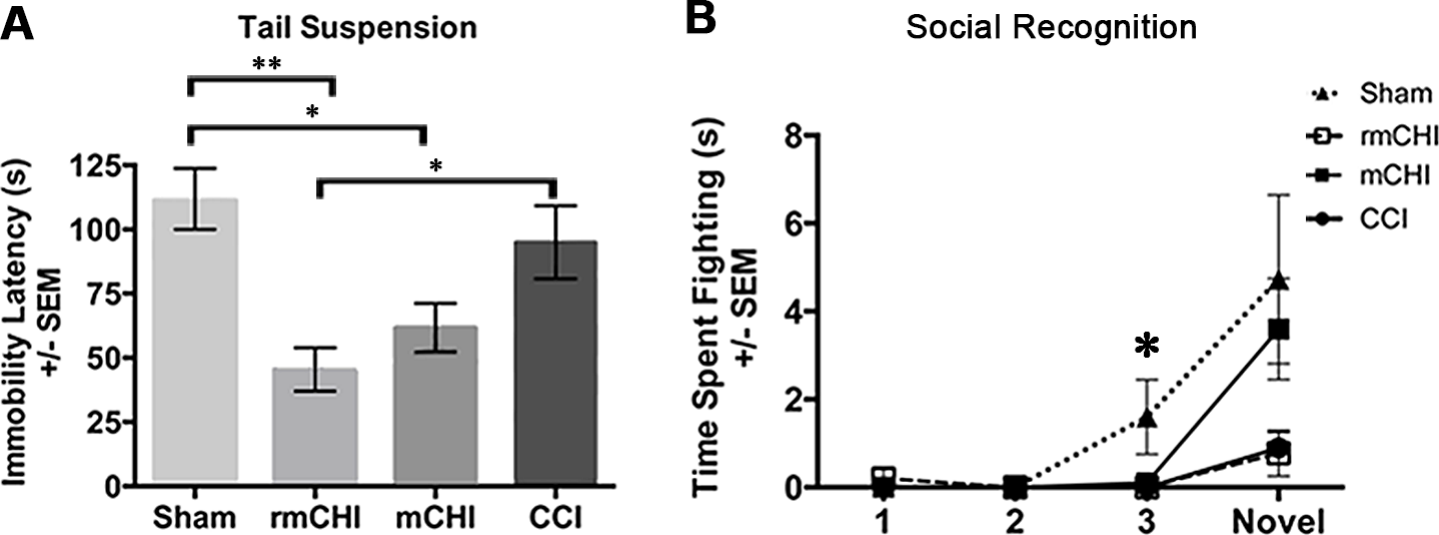
Tests of affective behavior at 90 dpi suggest that TBI induces depression and increased passivity towards other mice. **(A)** Depression-like behaviors were tested using the tail suspension test, in which both mCHI and rmCHI animals gave up (became immobile) more quickly than shams (**p<0.01 and *p<0.05, respectively), and rmCHI mice gave up more quickly than CCI animals (*p<0.05). Interestingly, CCI mice were not different than shams. **(B)** When aggressive behavior was assessed in a social recognition test, sham, but not injured, mice engaged in more fighting with the stimulus mouse by trial 3 (*p<0.05). rmCHI and CCI, but not mCHI, mice were more passive than shams when a new mouse was introduced on the 4^th^ (novel) trial, although not significantly.

The elevated zero maze test at 90 dpi assessed exploratory behaviors in an anxiety-provoking environment. Brain injured mice (mCHI, rmCHI, CCI) exhibited no differences in open quadrant entries, head dips, stretch and attend postures, or time spent in the enclosed quadrants (data not shown). Depression-like behaviors were tested with the tail suspension test (F_3, 35_ = 7.05, *p* < .01), in which both mCHI and rmCHI animals became immobile more quickly than shams (*p* < .05 and *p* < .01, respectively; Fig 6A), and rmCHI mice also became immobile more quickly than CCI animals (*p* < .05). Interestingly, CCI animals did not differ from shams. The forced swim task (which is often used to test similar behaviors in rats and is generally regarded to be less reliable in mice), revealed no statistically significant differences among the experimental groups (F_3, 35_ = 1.87, *p* = .15). However, rmCHI mice that gave up early on the tail suspension test also gave up earlier on the forced swim test (data not shown).

When behavior was assessed in a novel social interaction test, shams, but not injured, mice started to fight with the stimulus mouse by trial 3 (F_3, 35_ = 3.27, *p* < .05; Fig 6B). rmCHI and CCI, but not mCHI, mice were less aggressive and dominant than shams when a new mouse was introduced on the 4^th^ (novel) trial, though not statistically significant.

**Fig 7:**
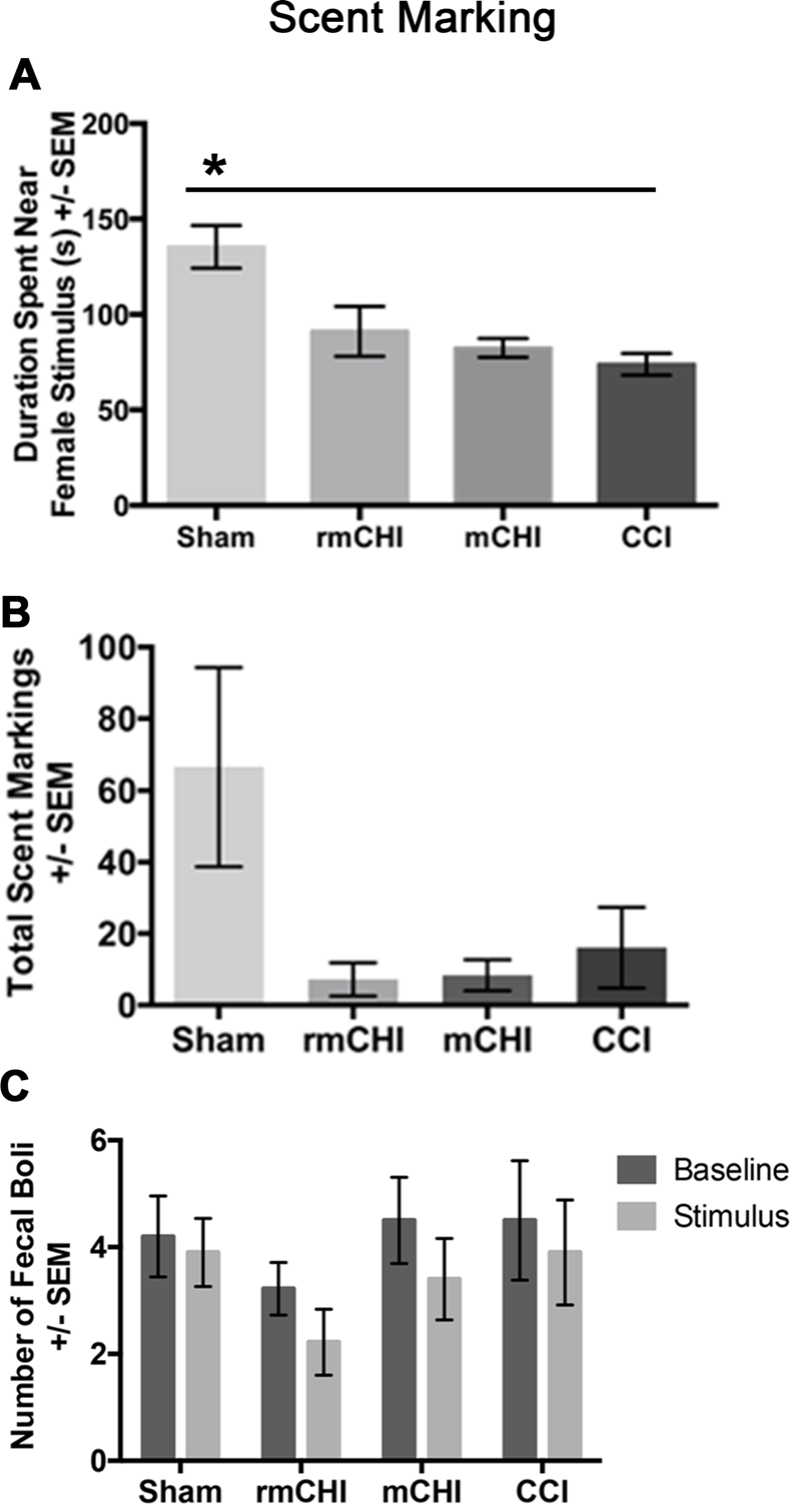
TBI leads to social inhibition. **(A)** All injured mice spent less time exploring near the female stimulus mice (*p<0.05). **(B)** rmCHI mice appeared to produce the least scent markings across injury groups, but results were not statistically significant. **(C)** There were no significant differences in the number of fecal boli produced by each animal across injury groups between stimulus and baseline testing.

In the scent-marking test, all injured groups spent less time exploring the novel female compared to the sham mice (F_3, 33_ = 16.03*p* < .01; Fig 7A). Injured mice also produced the least amount of scent markings compared to sham, though results did not reach statistical significance (F_3, 35_ = 1.47, *p* = .27; Fig 7B). There were no significant differences between the injury groups in the number of fecal boli during the baseline (F_3, 35_ = .50, *p* = .69) and stimulus (F_3, 35_ = 1.00, *p* = .40; with female mouse) trials (Fig 7C).

## Discussion

Animal models have been developed to better understand the pathology and consequences of trauma, particularly mild injuries. There are several rodent injury models of mild and moderate brain injuries, however few animal studies have shown the development and progression of affective disorders similar to those reported in clinical populations [24,35,46,47]. We report for the first time that a single mild CHI and repeated mild CHI (3 days apart) can lead to the presentation of depression-like behaviors as late as 90 days after injury. These findings show that motor deficits (turn bias while swimming) and affective abnormalities are present for at least several months after repeated concussive injury in mice.

CCI is commonly used to model the pathological and behavioral features of moderate to severe injury observed in human TBI patients. We have previously shown that mild CCI causes extensive tissue white matter disruption [38,39] and that moderate injuries cause motor and behavioral impairments [26,30,40], confirming our current findings. General activity levels are also prominently altered in CCI mice and rats. They appear to be more anxious and are less likely to engage in normal exploratory behavior. We have shown that rats with repeated mild CCI moved significantly less during open field testing up to 30 dpi [61], although these differences were not observed at 90 dpi in our study. Similar to our present results, these animals did not display spatial learning or performance deficits on standard water maze testing [61]. Some reports have shown significant learning and memory deficits post CCI, though these findings are only observed when tested shortly after the initial injury. One such study reported that rats with moderate CCI took longer to find the platform in the water maze compared to sham 7–14 days after injury [62]. These findings suggest that behavioral impairments are present immediately following a TBI, and our new results suggest that subtle spatial learning deficits continue through long-term testing (> 90 dpi) in CCI mice.

Animal models of concussive injury have been developed that use predominately the weight-drop and CCI models (for review, see Semple et al. [22]). In mice, repeated mild injury intervals of hours to weeks have been used, and it is apparent that behavioral deficits, particularly motor alternations, often present during the acute injury phase (<7d). In contrast, both repeated weight drop and CCI models have been reported to induce cognitive deficits that extend weeks and months after CHI [27,32,63]. Similar cognitive deficits were also present several days after injury in a weight drop model for mild and repeated injury [64] and in a motorized impact model for mild and repeated injury [27]. Recently, Yang et al. [65] used an injury model similar as our CCI mice to induce rmCHI in mice. Mice were injured in a stereotaxic frame and received extensive rmCHI (3.8 mm impact depth) that resulted in acute structural MRI and motor alternations, followed by long-term anxiety-related behavior and spatial learning deficits [65].

One unique aspect of our CHI model is that a mild impact is accompanied by a rotational component. In contrast to most CHI models that restrain the head, our model uses an unrestrained mouse that is positioned on top of a foam cushion. As injury is inflicted, the head moves away from the impactor, the force sustained at the site of impact decreases with the addition of rotational stress caused by the same head displacement. This combination of impact followed by rotation models the angular acceleration of many human TBIs better than those previously reported. An additional strength of our study is that, after the early neurological testing, animals did not undergo further testing until 90 dpi, unlike the repeated behavioral testing reported other studies [39,61]. It is this combination of time and a rotational component that we believe results in the behavioral/neurological abnormalities seen in our CHI and rmCHI mice. We were specifically interested in determining whether an abnormal psycho-social phenotype would emerge. We have recently reported significant tissue damage following second mTBI, including increased edema and extravascular blood in the early post-injury phase [38], and ongoing white matter damage at a later time point (60 days) after repeated mild CCI [39]. The pathology that occurs after a diffuse rmTBI is likely to be different from other mTBI models, and our results suggest that mild brain injuries can lead to long-term behavioral deficits.

To our knowledge, the development of abnormal affective behaviors resulting from mild TBI 3 days apart has not been previously assessed at >90 dpi in rodents. In this study, we have shown affective and social behavioral deficits several months after rmCHI. Mice manage their territorial aggressiveness and communication through context-dependent social recognition, as indicated by increased urinary pheromone [59,66] and fecal boli markings [67]. Typically, dominant mice exhibit these behaviors in the presence of other mice. We have recently reported reduced scent marking behaviors in response to a novel female stimulus at adulthood after pediatric CCI (p21) [36]. Consistent to what we have reported previously, we found that rmCHI spent less time exploring and scent marking near female stimulus mice, suggesting that repeated head trauma could lead to passive and anti-social behaviors. Interestingly, both CCI and rmCHI mice were less aggressive towards a novel male mouse compared to shams. Brain injured rats in a weight drop model were also found to be passive towards a novel rat [68], however, younger mice with TBI may be more vulnerable to social effects, with deficits in social interaction and increased aggression leading into adulthood [67]. These results suggest that a diffuse and concussion-type of brain injuries and disturbance in affective behaviors may share potential common underlying mechanisms.

In brain-injured rodents, lesion location is a factor that contributes to social and affective outcomes, with injury to the frontal lobes and right hemisphere in particular being associated with behavioral deficits [69]. In our study, the CCI mice were impacted directly on the motor cortex while CHI mice were impacted on the skull directly above the frontal parietal region. The localized nature of CCI leads to predictable and distinct motor deficits as we and others have reported [25,40]. In contrast, the diffuse nature of CHI leads to greater axonal injury over a large brain region, including the anterior cortex [70]. Alterations in cell function such as excitotoxicity, calcium up-regulation, depolarization, and vulnerability of the blood brain barrier are components of the secondary response that continue to promote global brain damage [71,72]. In a midline fluid percussion injury model, diffuse injury leads to the sensitization of microglia and the inflammatory system, which have been suspected as an underlying mechanism for depressive-like behavior found at 7 dpi [34]. Microglia also remained primed and reactive up to 30 dpi, as a result of the prolonged secondary injury response [34].

Widespread damage to white matter and subsequent connectivity disruption lead to cognitive and neuropsychological impairments [73]. The rotational and more diffuse nature of our rmCHI model appears to lead to the presentation of depression-like behaviors. The tail suspension test is standard for measuring depression-like behaviors in mice by measuring the latency to stop struggling in an inescapable situation. In our study and others [24,55], injured mice gave up on the task quicker and spent more time immobile than shams, suggesting marked depression. Indeed, rmCHI mice were even more “depressed”, albeit not significantly more than the single mCHI group. These findings are further strengthened by the observation that there were no differences in general activity and anxiety-like behaviors that could confound our results. Changes in mood may have occurred in response to neuroinflammation, oxidative stress, and apoptosis, leading to increased cytokine production such as that typically associated with clinical depression [74–76]. Brain injuries from radiation [55] and stroke [77] also lead to depression-like behavior and social inhibition in animals [67]. Our data suggest that the incidence of depression emerges in rmCHI because of the brain’s increased vulnerability to the initial insult, wherein the subsequent mCHI further exacerbated the brain’s sensitivity to injury and contributed to the observation of behavioral deficits.

### Conclusions

In this study, we hypothesized that rmCHI would result in significant long-term cognitive deficits. Surprisingly, we found only subtle motor deficits (turn bias while swimming exhibited as a slight left sided hemiparesis) at 90 dpi that were similar to those observed in moderate CCI. Our findings support our hypothesis, in that the diffuse nature of mCHI and rmCHI led to the development of depressive-like behaviors observed at 90 dpi without overt motor deficits. Further investigations are necessary to explore the mechanistic pathways underlying the emergence of depression in rmCHI and to develop therapeutics and/or complementary dietary treatments to alleviate these behaviors.
